# Life of Dr. John Ellis Marshall, Being a Report Made by a Committee Appointed for This Purpose by the Buffalo City Medical Association

**Published:** 1850-10

**Authors:** J. Trowbridge, Frank H. Hamilton

**Affiliations:** Committee; Committee


ART. II.—*Life of Dr. John Ellis Marshall, being a report made by a Committee appointed for this purpose by the Buffalo City Medical Association.* Communicated by resolution of the Association.

Gentlemen,—The Committee appointed by a resolution of this Society to prepare a brief Biography of the late Dr. John Ellis Marshall, would state, that the delays and embarrassments naturally incident to the performance of a duty of this kind, have prevented the possibility of an earlier report. Indeed, so many years have elapsed since the death of Dr. Marshall, and so few of his cotemporaries now remain among us, we feel confident that many circumstances of his useful life have escaped our notice. While we regret this loss, and the more especially because it renders this intended tribute of respect less complete, and deprives us all of much that was doubtless worthy of imitation in his example, it ought to admonish us that hereafter if we would desire to perpetuate the lives and services of our deceased co-laborers, we must secure the record before time has drawn over them her impenetrable veil.

John Ellis Marshall, the only child of Thomas and Sarah Marshall, was born March 18th, 1785, in Norwich, New London County, Connecticut. His mother dying when he was only one month old, he was adopted into the family of Daniel Ellis, of Franklin, Connecticut, and educated by him as one of his own children.

At the age of sixteen years he was attacked with a pulmonary affection which required him to spend a year or more abroad for his health. When he returned he was so far recovered as to enable him to resume his studies of the higher branches of the English, and of the classics, under the tuition of the Rev. Dr. Samuel Nott, of Franklin, a brother of Dr. Eliphalet Nott, President of Union College. His venerable and distinguished preceptor is still living and preaching, at the age of ninety years and upwards, in the same county in which he then lived.

He commenced his medical studies when twenty years old, under the instruction of Dr. Philemon Tracy, an eminent practitioner, of Norwich, and father of the Hon. A. H. Tracy, late member of Congress from Western New York. Dr. Peter Allen, of Kinsman, Ohio, a polished gentleman, and a physician of distinguished reputation, informs the writer, that Dr. Marshall commenced the study of medicine on the same day, and in the same office with himself: and that they remained together during their three years of pupilage, and were licensed to practise at the same time, on the third day of August, 1808.

"Many changes having since taken place in the science of medicine," says Dr. Allen, "I will mention our course of reading and the principal authors in each branch. On Anatomy we read Chesselden; on Physiology, Haller; on Theory and Practice of Medicine, Cullen, Boerhaave, Brown, Darwin, and Townsend; on Surgery, Benjamin Bell, with De Sault on Fractures and Dislocations; on Obstetrics, Denman; on Chemistry, Lavoisier. We read also Cullen's Materia Medica, and the Dispensatories of the day." "Changes," indeed, have occurred! Such as while they clearly indicate the rapid advance of our science, must also serve to illustrate the brevity and vanity of the most exalted literary reputation. It is scarcely half a century since these authors were ultimate and often sole authority in the several departments in which they wrote, and now not one of them all can be regarded as standard, or will make any pretensions to authority when not sustained by the numerous, and *perhaps* wiser generation which has succeeded them. Like venerable Fathers, they repose at ease upon our shelves, objects of love and respect, but of whose opinions upon most matters of importance we are wont to say, and possibly without having ever consulted them, "they are much behind the age."

"Dr. Tracy," continues Dr. Allen, "gave the most unwearied attention to our studies, and frequently took one of us with him in his visits to the sick; and it is presumed that Dr. Marshall would have attributed much of his success in after life to the attention and instruction of our excellent preceptor."

"Two years of the three in which we were together, we occupied the same room and bed, and of course I had a good opportunity to become acquainted with the character of Dr. Marshall. As a student, he was thorough and industrious; his judgment sound and discriminating, and he passed over nothing lightly, nor without a clear and complete comprehension of its meaning. Although not then a professor of religion, he was conscientious in the discharge of what he considered duty, and he scrupulously avoided what he believed was wrong. His manners were conciliating, and his conduct always marked by discretion. I do not know that he ever made an enemy, or gave momentary offence."

This is the testimony of one who knew him, at that time, more intimately than any other person now living, and who thus early, in the character and habits of the student, was able to predict the future respectability and success of the man. Integrity and talent devoted to any particular pursuit, is an investment from which the owner never fails, sooner or later, to receive profitable dividends.

Having been examined by the Censors of the County of New London, Conn., as has been already stated, with his friend Dr. Allen, in August, 1808, he was declared qualified, and was licensed to practise medicine and surgery under the diploma and seal of the "Connecticut Medical Society." A few months after, he opened an office in Oxford, Chenango county; but although his success and prospects were exceedingly flattering, he soon became dissatisfied, and tempted by the fertility and rapid settlement of the western portion of the State, he removed, in October following, to Mayville, Chautauque county, where he resumed and continued the practice of his profession with eminent success, for six years. Here, also, he was married, in September, 1810, to Ruth, daughter of Orsamus Holmes, Esq., a gentleman of high respectability, and one of the earliest pioneers of Chautauque county. Mrs. Marshall has survived her husband, and still lives among us, esteemed and respected no less for the many feminine virtues which adorn her character, than for the rank once occupied by her deceased and lamented partner.

Having received an appointment as Surgeon to the Second Regiment of the New York State Militia, on the 15th of April, 1812, during the war with Great Britain, he was ordered by Col. McMahan, Dec. 20, 1813, to join the regiment at Buffalo, to which he promptly responded. After about five months service on this frontier, his regiment was disbanded and he returned home. But on the 1st of August, 1814, he was again ordered to report himself speedily, the next day, at Buffalo, at the house of Mr. Perry, the old rendezvous of his regiment. The season and the locality, together with the privations and exposures of a camp life, now conspired to produce among both officers and privates of the regiment a great amount of sickness, especially fevers, diarrheas and other similar diseases incident to miasmatic localities. During the whole of August and September, the hospitals were crowded, and many went home on limited furloughs. Upon Dr. Marshall alone, as senior officer, devolved a great portion of the hospital duties, and after about four weeks of unremitting toil, worn down with labor and care, he also fell sick, and removed on a furlough of twelve days to his home in Mayville. He did not return, however, for several weeks, and he was even then in a very feeble state of health, yet he resumed his duties and continued at his post until in the fall following, when his regiment was discharged. It is believed that for many years afterwards he suffered in the spring and fall from a recurrence of biliary derangements, the origin of which was distinctly traceable to this campaign.

On the first organization of Chautauque county, in February, 1811, Dr. Marshall was appointed under Daniel D. Tompkins, then Governor of this State, County Clerk; which office he held, and the duties of which he continued to perform, except when absent with his regiment, until his removal to Buffalo in March, 1815.

It was in this latter place, where a wider field was presented for the display of his talents, and for the appreciation of his skill, that he arose to the highest distinction in the ranks of his profession. No man has acquired in our city, a more solid and extensive reputation as a practitioner of medicine, nor a more enviable name as a citizen. As evidence of the first, the writer can refer to the universal testimony of those who were his cotemporaries, and who still survive him; by *all* of whom, we believe, his memory is held in respect, and his example presented as worthy of imitation. Nor can we avoid arresting attention to this fact, as proof, if proof is needed, that elevated rank in our profession does not necessarily invoke upon its possessor either the jealousy or enmity of his brethren, and that no one receiving the continued reproofs and censures of his fellow-cititizens and professional rivals may cherish this as welcome evidence, that he has either talent, integrity, reputation, or any other truly enviable distinction. As evidence of the estimate placed upon his professional attainments abroad, we may mention that he was made an honorary member of the "Medical Society of the City of New York," and also in 1838, of the "Medical Society of the Geneva College," as a mark, writes the Secretary, "of the respect in which your professional reputation is held by the members of this Society."

Dr. Marshall received also, at different times, various appointments under the State and municipal governments, which sufficiently indicate his position as a citizen. In February, 1818, he was appointed Clerk of Niagara county, then embracing the present county of Erie. He held, also, in 1832, the office of Health Physician to the city.

On Saturday evening, Dec. 2, 1838, Dr. Marshall was attacked with the sickness which, on the following Thursday, terminated fatally. His disease was a pleuro-pneumonitis, and its progress was both rapid and severe, being accompanied in most of its course with great physical suffering. Yet his death was calm and cheerful. He died with the dignity of a man, and the confidence of a Christian.

In reviewing the character of Dr. Marshall, with a view to present a summary of his most striking traits, it has occurred to us that it will be comprised in a single extract from the discourse preached at his funeral, by the late Rev. Dr. Hopkins, which we shall therefore quote:

"Time would fail me were I to attempt to dwell on the habits of integrity, punctuality and scrupulous exactness, which distinguished him in all the business of life. And while these severer virtues are mentioned, it may not be forgotten that his heart was the seat of the more soft and winning virtues. Amiable and kind in the social circle, he gathered the full confidence and affectionate esteem of those to whom he became known.

"Perhaps I cannot better complete the sketch than by saying that his character possessed great *harmony* and *consistency*. It is frequently the case that some one trait is cultivated at the expense of another; so that, in the same individual one excellence is more marked by its prominence above others, or by its contrast with them. In Dr. Marshall, however, the chief elements of a good character as a man and a citizen were admirably blended. And, as when the different colored rays of light combined in their proper relations and proportions constitute that mellow ray which is at once beautiful and useful, so in him, those several traits which compose the character of the good citizen and useful man seemed to be so harmoniously blended that it would be difficult to distinguish their boundaries. Firmness and candor, seriousness and cheerfulness, humility and dignity, not only appeared in him to be reconcilable virtues, but each appeared to reflect a glory on the other. These produced great symmetry of character, and great evenness of conduct."

Committee, { J. Trowbridge,

Frank H. Hamilton.

